# 90 Nakajo-Nishimura syndrome: a case report

**DOI:** 10.1093/rheumatology/keac496.086

**Published:** 2022-10-07

**Authors:** Zerguine Halima, Djohra Hadef, Hanane Soltani, Younes Zerrouki, Sana Ouazani, Hafidha Belkacemi, Safia Challa, Samy Slimani

**Affiliations:** 1 Department of Pediatrics, CHU Batna, Algeria; 2 Department of Nephrology, CHU Batna, Algeria; 3 Atlas Clinic of Rheumatology, Batna, Algeria

## Abstract

**Introduction:**

Nakajo-Nishimura is a rare autosomal recessive inherited autoinflammatory condition that has been described mainly in the Japanese population, but very similar syndromes, such as Candle syndrome, have been reported in Western populations. There is no gold standard treatment for this condition. This is a case report of a 12-year-old female who most likely has Nakajo-Nishimura syndrome.

**Methods and results:**

A 12-year-old female (born in 2010), from Batna (Algeria), was born of a first-degree consanguineous marriage, and the second of two children. She had no relevant past medical history. She was referred on account of dermatology symptoms, occurring very early in life, with papules and distal purplish erythema located in fingers and toes associated with an acrocyanosis. Subsequently, diffuse arthralgias appeared, exacerbated by cold, with a delay in walking, around the age of 3 years.

Clinical examination revealed a thread-like appearance of the fingers with shortening of the 5^th^ digit, and small erythematous lesions, without sclerosis or Raynaud's disease. There was no sign of synovitis or joint deformities. Hepatosplenomegaly with dental dysgenesis and delayed puberty were noted. The laboratory tests showed an inflammatory syndrome—ESR (Erythrocyte Sedimentation Rate) of 100 mm the first h, positive CRP (C-reactive protein), hyper alpha and gamma globulinaemia, and inflammatory anaemia with thrombocytopaenia. The genetic work-up is currently in progress.

The treatment was Hydroxychloroquine and steroids. After the onset of a nephrotic syndrome, Mycophenolate mofetil (MMF) was added. The renal biopsy found a renal amyloidosis. The patient was in chronic renal failure stage 4 requiring hemodialysis.

**Conclusion:**

Extremely rare syndrome described in 30 patients in the literature and whose treatment remains symptomatic and does not prevent complications.

**Disclosure of Interest:**

None declared

